# Inferring Latent Disease-lncRNA Associations by Label-Propagation Algorithm and Random Projection on a Heterogeneous Network

**DOI:** 10.3389/fgene.2022.798632

**Published:** 2022-02-04

**Authors:** Min Chen, Yingwei Deng, Ang Li, Yan Tan

**Affiliations:** Hunan Institute of Technology, School of Computer Science and Technology, Hengyang, China

**Keywords:** disease similarity, lncRNA similarity, space projection, computational prediction model, label-propagation algorithm

## Abstract

Long noncoding RNA (lncRNA), a type of more than 200 nucleotides non-coding RNA, is related to various complex diseases. To precisely identify the potential lncRNA–disease association is important to understand the disease pathogenesis, to develop new drugs, and to design individualized diagnosis and treatment methods for different human diseases. Compared with the complexity and high cost of biological experiments, computational methods can quickly and effectively predict potential lncRNA–disease associations. Thus, it is a promising avenue to develop computational methods for lncRNA-disease prediction. However, owing to the low prediction accuracy ofstate of the art methods, it is vastly challenging to accurately and effectively identify lncRNA-disease at present. This article proposed an integrated method called LPARP, which is based on label-propagation algorithm and random projection to address the issue. Specifically, the label-propagation algorithm is initially used to obtain the estimated scores of lncRNA–disease associations, and then random projections are used to accurately predict disease-related lncRNAs.The empirical experiments showed that LAPRP achieved good prediction on three golddatasets, which is superior to existing state-of-the-art prediction methods. It can also be used to predict isolated diseases and new lncRNAs. Case studies of bladder cancer, esophageal squamous-cell carcinoma, and colorectal cancer further prove the reliability of the method. The proposed LPARP algorithm can predict the potential lncRNA–disease interactions stably and effectively with fewer data. LPARP can be used as an effective and reliable tool for biomedical research.

## Introduction

Long noncoding RNAs (lncRNAs) are more than 200 nucleotides long and lacks protein-coding RNAs (Peng et al., 2019). Studies have shown that lncRNAs are closely related to biological processes such as chromatin modification, transcription, translation, splicing, and epigenetic regulation (Wang and Chang, 2011; Wapinski and Chang, 2011; Song et al., 2014; Sun et al., 2017; Tian et al., 2021; Peng et al., 2021). The abnormal function of lncRNAs can reportedly lead to abnormal cell behavior, and lncRNAs are related to the occurrence and development of many human diseases. For example, Wang et al. [5] found that lncRNA PVT1 promotes the progression of melanoma through endogenous sponge cell miR-26b, and Cai et al. (2018) found that BCAR4 can activate the GLI2 signaling pathway in prostate cancer. The specific secondary structure of lncRNAs and its ability to control gene expression also render it an ideal target for drug development (Chen ZJ. et al., 2016; Tripathi et al., 2018; Xu et al., 2019). Our current understanding of the role of lncRNAs in disease is far from complete, so further understanding the relationship between lncRNAs and diseases is significant. However, experimentally identifying the association between lncRNAs and diseases through biotechnology is expensive and laborious. Increased attention is being paid to predicting the association between lncRNAs and diseases by computational prediction method.

Many researches predicted the associations between lncRNAs and diseases based on known information about lncRNA–disease associations, disease–disease similarity information, and lncRNA–lncRNA similarity information. Based on the hypothesis that similar diseases may be related to lncRNAs with similar functions, many researches used information such as lncRNA–disease association network, disease-similarity network, and lncRNA similarity network to realize the association prediction between lncRNAs and diseases through random-walk algorithm. For example, Sun et al. (2014) constructed a random-walk model RWRlncD based on global network, but this method cannot be used to predict isolated diseases (diseases without any lncRNA associated with it). Chen X. et al. (2016) proposed an improved prediction model with restart random-walk algorithm (RWR), IRWRLDA. Yu et al. constructed a prediction model based on double random walk (Yu et al., 2017). Li et al. (2019c) developed an improved local random-walk prediction model, LRWHLDA. Fan et al. (2019) combined positive-point mutual information with multiple heterogeneous information and then implemented RWR to construct an lncRNA–disease correlation prediction model IDHI–MIRW, Li et al. (2019b) constructed an lncRNA-disease-associated prediction model, TCSRWRLD, by using node information called as target convergence set combined with random-walk algorithm, but the prediction accuracy of these methods is not very high.

Chen (2015a) applied KATZ index to lncRNA–disease association prediction, and this model can infer potential lncRNAs without known related diseases. Ping et al. (2018) used known lncRNA–disease associations to construct a binary network and then predicted the lncRNA–disease association based on its strict power-law distribution. According to the path length in the lncRNA–disease heterogeneous network, Xiao et al. (2018) predicted the probability of lncRNA–disease association. Liu et al. (2019) constructed a weighted network based on the resource-allocation strategy of unequal allocation and unbiased consistency and then applied the label-propagation algorithm to predict the lncRNA–disease association. However, the prediction results of these methods may be biased toward lncRNAs with more known related diseases and diseases with more known related lncRNAs.

With the rapid development of machine-learning technology, many researches used machine-learning methods to predict potential lncRNA–disease associations and miRNA-disease-associated prediction (Liang et al., 2019). For example, Yu et al. (2018); Yu et al. (2019) proposed two prediction models based on the Naïve Bayes classifier to infer potential lncRNA–disease associations. Guo et al. (2019b) used autoencoder neural network and Rotating Forest to predict the associations between lncRNAs and diseases. Liang et al. (2021) identified cancer subtype by using graph autoencoders. Chen et al. (2018); Chen X. et al. (2019) predicted miRNA-disease association by using the decision-tree model. Zhao et al. (2019) predicted miRNA-disease association by using adaptive boosting. Chen et al. (2017a) predicted miRNA-disease association by using support vector machine combined with k-nearest neighbor method. In this type of machine-learning prediction model, the main disadvantage is that negative samples are required as a training set. Given that negative samples are usually difficult to obtain, their prediction performance is significantly affected. Many semi-supervised methods are attracting attention. Xuan Z. et al. (2019). developed a probabilistic matrix-factorization model based on semi-supervised learning methods to identify potential associations between lncRNAs and diseases. Laplacian regularized least squares obtained wide application in the area of bioinformatics (Shen et al., 2021). By fusing the semantic similarity and cosine similarity of disease, lncRNA expression similarity, and cosine similarity. Lan et al. (2020) denoised lncRNA feature information and disease feature information with an automatic encoder. They then predicted lncRNA-disease association by using matrix-decomposition algorithm. Xie et al. (2019) predicted the association between lncRNAs and diseases by Laplacian regularized least squares. Chen et al. (Chen and Yan, 2013) developed a model LRLSLDA that uses Laplacian regularized least squares to identify the associations between lncRNAs and diseases. Later, on the basis of LRLSLDA, Chen et al. (2015) proposed a new lncRNA–disease association prediction model, LRLSLDA–LNCSIM. Huang et al. (2016) used the topological feature of a directed acyclic graph of disease-similarity network to propose another improved model ILNCSIM. None of these semi-supervised methods require negative samples to train the model, but the problem of how to select parameters more reasonably has not been resolved.

In recent years, deep learning has attracted increased attention from artificial intelligence communities (Lihong et al., 2021; Zhou et al., 2021a; Lihong et al. (2021) and Zhou et al. (2021b) developed two deep learning-based models, deep Learning framework with Dual-net Neural Architecture and multiple-layer deep model based on gradient boosting decision trees, to predict possible lncRNA-protein interactions. Xuan et al. proposed a series of lncRNA–disease association prediction models based on convolutional neural networks, including CNNLDA (Xuan et al., 2019a), GCNLDA (Xuan et al., 2019c), CNNDLP (Xuan et al., 2019d), and LDAPred (Xuan et al., 2019b). Wu et al. (2020) also predicted the potential association between lncRNA and disease by using a graph-convolutional network. Lan et al. (2021) denoised heterogeneous data through principal component analysis. They then extracted features by graph-attention network, ultimately predicting the potential association between lncRNA and disease by using multilayer perceptron. These methods have good performance in lncRNA–disease association prediction, but the parameters of these models are relatively difficult to determine.

Various biological information from different sources can help us understand the relationships between lncRNAs and diseases more comprehensively (Chen et al., 2017b; Fu et al., 2018) (Peng et al., 2017). For example, Liu et al. (2014) integrated the human lncRNA expression profile, human gene-expression profile, and other data to predict lncRNA–disease association. This method can achieve lncRNA–disease association prediction without knowing lncRNA–disease association. Chen Q. et al. (2019) used support-vector machine (SVM) to implement lncRNA–disease association prediction by integrating lncRNA–gene interaction, lncRNA–disease association, and disease semantic similarity. Lu et al. (2018) integrated known lncRNA–disease interactions, disease–gene interactions, and gene–gene interactions and used the inductive matrix-completion method to identify the associations between lncRNAs and diseases. Ding et al. (2018) combined gene–disease and lncRNA–disease association information and established a lncRNA–disease association prediction model, TPGLD, based on a lncRNA–disease–gene tripartite network. Wang Y. et al. (2019) pre-set weights for various association matrices between genes, lncRNAs, and diseases, decomposed these matrices into low-rank matrices, and developed a weighted-matrix decomposition lncRNA–disease association prediction model WMFLDA. Chen (2015b) predicted lncRNA–disease association through the integration of lncRNA–miRNA interaction and miRNA–disease correlation. Zhang et al. (2019) developed a prediction model based on DeepWalk through the integration of miRNA–disease, lncRNA–disease, and miRNA–lncRNA correlation. Zhou et al. (2015) realized the random-walk algorithm on the heterogeneous network composed of the known lncRNA–disease-related network, miRNA-related lncRNA crosstalk network, and disease-similarity network and proposed a prediction model, RWRHLD. Wang et al. (2016) used the known lncRNA–miRNA crosstalk to develop a sequence-based lncRNA–disease association prediction model, LncDisease. However, owing to the high false negatives and positives in the prediction of miRNA–lncRNA interaction, the performance of LncDisease is limited.

Zhao et al. (2015) integrated genome, transcriptome, and rule set data and then used the naïve Bayesian classifier to predict the lncRNA–cancer association. Lan et al. (2016) integrated information such as lncRNA sequence information, disease–gene associations, and GO annotations and identified new lncRNA–disease associations through bagging SVM. Fu et al. (2018) used different biological data sources of lncRNAs, miRNAs, genes, disease, and drugs for prediction, decomposed the correlation matrix into different biological entities, and reconstructed the lncRNA–disease correlation matrix through matrix decomposition. However, the method does not deal with the noise of the original features, so the prediction performance is not high. Sumathipala et al. (2019) integrated integrin disease, protein–lncRNA, and protein–protein correlation and used the network-diffusion method to predict lncRNA–disease association. Zhang et al. (2018) used lncRNA similarity, protein–protein interactions, and disease similarity to construct a composite network and then used flow-propagation algorithm for prediction. Guo et al. (2019a) constructed a molecular-association network based on the known association among diseases, proteins, miRNA, lncRNA, and drugs and then used random-forest classifier to infer the association between any two of them. The above studies can help elucidate cellular processes and complex pathogenesis at the molecular level to a certain extent, but the use of multiple biological data sources may introduce noise and irrelevant information, leading to increased false-positive rates.

In the present study, we proposed an lncRNA–disease association prediction method called LPARP, which is based on a label-propagation algorithm and random projection. LPARP uses the semantic similarity of diseases, functional similarity of lncRNAs, and known information on lncRNA–disease association and then predicts them through label-propagation algorithms and random projections. Experimental results showed that LPARP is superior to several existing classic methods in predicting candidate lncRNAs. Case studies on bladder cancer, esophageal squamous-cell carcinoma, and colorectal cancer show that LPARP can effectively identify potential diseases associated with lncRNAs.

## Materials and Methods

### Materials

#### LncRNA-Disease Association Network

Known experiments supporting lncRNA–disease-related data are from the lncRNADisease database (Chen et al., 2012). We obtain three datasets of lncRNA–disease-related data from different versions of the database. From the 2014 version, 352 pairs of lncRNA–disease-related data are obtained, covering 156 lncRNAs and 190 diseases (dataset1); from the 2015 version, 621 pairs of associations are obtained, covering 285 lncRNAs and 226 diseases (dataset2); from the 2017 version, 1,695 pairs of associations are obtained, including 828 lncRNAs and 314 diseases (dataset3). For convenience, a Boolean matrix $LD={\left(ld_{ij}\right)}_{nl\times nd}$ is used to represent the association between lncRNAs and diseases. If a known association exist between lncRNA l_i_ and disease d_j_, then LD (i,j) = 1. Otherwise, LD (i,j) = 0. n_l_ and n_d_ are used to represent the number of diseases and lncRNA, respectively.

### Disease Semantic Similarity

Many researchers used disease semantic similarity data to describe the similarity between diseases. In this method, the disease is represented as a directed acyclic graph (DAG), and then the similarity between the diseases is calculated based on the DAG. The detailed calculation process can be found in literature (Wang et al., 2010). This method is used to calculate the semantic similarity between diseases, as represented by matrix DD.

### LncRNA Functional Similarity

Considering that lncRNAs with similar functions are often associated with similar diseases, we calculate the functional similarity between diseases based on the semantic similarity of diseases. This type of method is used in many lncRNA–disease associations (Chen et al., 2020; Zhang et al., 2020; Zhang et al., 2021). It will not be introduced in detail here. The matrix LL is used to represent the functional similarity of lncRNA.

### Disease (LncRNA) Gaussian Interaction-Profile Kernel Similarity

Many zeros exist in the disease semantic similarity matrix DD and the lncRNA functional similarity matrix LL, so we further introduce the Gaussian interaction-profile kernel similarity (van Laarhoven et al., 2011) to improve this shortcoming. The Gaussian interaction-profile kernel similarity is also based on the assumption that lncRNAs with similar functions are often associated with diseases with similar phenotypes. The Gaussian interaction-profile kernel similarity between lncRNAs is defined as follows:

$$GL\left(i,j\right)=\exp\left(-\gamma_{l}{\left\|lp\left(l_{i}\right)-lp\left(l_{j}\right)\right\|}^{2}\right)$$ (1)

$lp\left(l_{i}\right)$ indicates the number of diseases associated with lncRNA $l_{i}$ , and $\gamma_{l}$ is the width of the nuclear spectrum, defined as follows:

$$\gamma_{l}=\frac{1}{\frac{1}{nl}\sum_{i=1}^{nl}{\left\|lp\left(l_{i}\right)\right\|}^{2}}$$ (2)

Similarly, we can obtain the similarity of Gaussian nuclear spectrum between diseases:

$$GD\left(i,j\right)=\exp\left(-\gamma_{d}{\left\|lp\left(d_{i}\right)-lp\left(d_{j}\right)\right\|}^{2}\right)$$ (3)

$lp\left(d_{i}\right)$ is the number of lncRNAs associated with disease $d_{i}$ , and $\gamma_{d}$ is the width of the nuclear spectrum, defined as follows:

$$\gamma_{d}=\frac{1}{\frac{1}{nd}\sum_{i=1}^{nd}{\left\|lp\left(d_{i}\right)\right\|}^{2}}$$ (4)

### Integrated Disease Similarity and lncRNA Similarity

Next, lncRNA functional similarity and lncRNA Gaussian interaction-profile kernel similarity are integrated to construct lncRNA similarity.

If the functional similarity between lncRNA node $l_{i}$ and lncRNA node $l_{j}$ is 0, the similarity between $l_{i}$ and $l_{j}$ is taken as the lncRNA Gaussian interaction-profile kernel similarity value between $l_{i}$ and $l_{j}$ . Otherwise, the value is the functional similarity LL between $l_{i}$ and $l_{j}$ , and the formula is as follows:

$$LL^{f}\left(i,j\right)=\{\begin{array}{c} LL\left(i,j\right)\\ GL\left(i,j\right) \end{array}\begin{array}{c} ,\;if\;LL\left(i,j\right)\neq 0\\ ,\;otherwise \end{array}$$ (5)

In the same way, the semantic similarity between diseases and the Gaussian interaction-profile kernel similarity between diseases are used to construct the similarity between diseases.

$$DD^{f}\left(i,j\right)=\{\begin{array}{c} DD\left(i,j\right)\\ GD\left(i,j\right) \end{array}\begin{array}{c} ,\;if\;DD\left(i,j\right)\neq 0\\ ,\;otherwise \end{array}$$ (6)

### LDAI-ISPS Workflow Model

**FIGURE 1:**
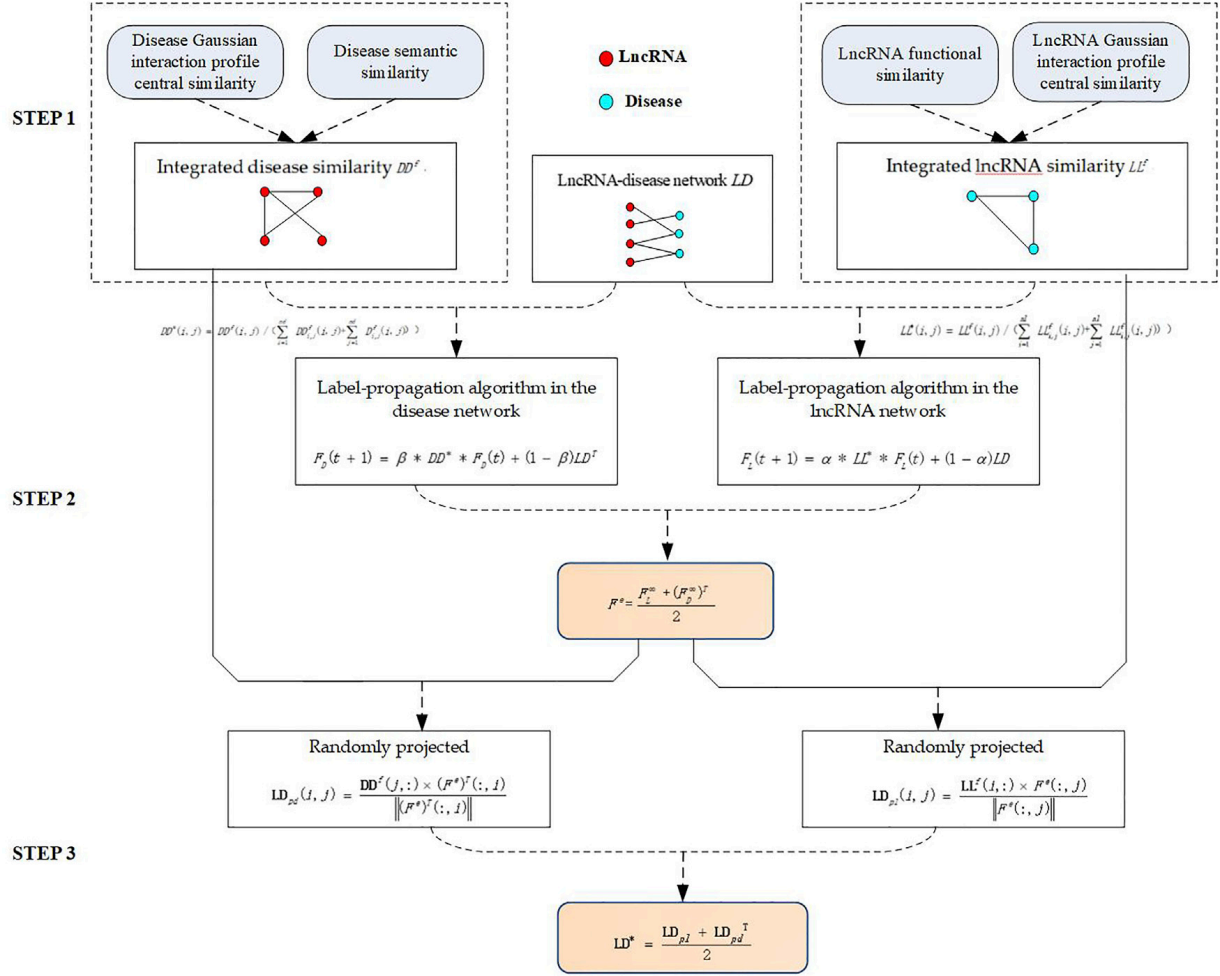
The flowchart of LPARP.

The algorithm is divided into three steps. In step 1, Integrated disease similarity is constructed by using semantic similarity between diseases and the Gaussian interaction-profile kernel similarity between diseases, and integrated lncRNA similarity is constructed by using functional similarity between lncRNAs and Gaussian interaction profile kernel similarity between lncRNAs.In step 2, the label-propagation algorithm is used to obtain the estimated score of lncRNA–disease association. In step 3, random projections are used to obtain precise scores of lncRNA–disease associations. (Figure 1.).

#### Estimated Score of lncRNA–Disease Association

First, the label-propagation algorithm in the lncRNA network is implemented. During the label-propagation process, each point retains the information of its neighbors and receives its initial label information. The iterative equation can be written as follows (Wang and Zhang, 2008):

$$F_{L}\left(t+1\right)=\alpha *LL^{*}*F_{L}\left(t\right)+\left(1-\alpha \right)LD$$ (7)

In the above formula, t represents the time step, $F_{L}\left(t\right)$ represents the result of the *t*th iteration in the label-propagation algorithm, and LD is the known lncRNA–disease association matrix, which represents the initial matrix. α ∈[0,1] is a hyper-parameter used to balance the ratio between the information from its neighbors and its initial label information. $LL^{\text{*}}$ is the normalized matrix of the integrated lncRNA similarity network $LL^{f}$ , whose calculation method is as follows:

$$LL^{*}\left(i,j\right)=LL^{f}\left(i,j\right)/\left(\sum_{i=1}^{nl}LL_{i,j}^{f}\left(i,j\right)+\sum_{j=1}^{nl}LL_{i,j}^{f}\left(i,j\right)\right)$$ (8)

After finite iterations, the probability space reaches a stable state $F_{L}^{\infty }$ ( $\left|F_{L}\left(t+1\right)-F_{L}\left(t\right)\right|<10^{-6}$ )to stop the iteration.

Then, the iterative equation of the label-propagation algorithm in the disease is implemented as follows:

$$F_{D}\left(t+1\right)=\beta *DD^{*}*F_{D}\left(t\right)+\left(1-\beta \right)LD^{T}$$ (9)

$LD^{T}$ is the transposed matrix of LD . Let $F_{D}\left(0\right)=LD^{T}$ . β ∈[0,1] is a hyper-parameter used to control the rate of retaining information from neighbors and $DD^{\text{*}}$ is the normalized matrix of the integrated disease-similarity network $DD^{f}$ , then the calculation method is as follows:

$$DD^{*}\left(i,j\right)=DD^{f}\left(i,j\right)/\left(\sum_{i=1}^{nd}DD_{i,j}^{f}\left(i,j\right)+\sum_{j=1}^{nd}D_{i,j}^{f}\left(i,j\right)\right)$$ (10)

The prediction result of the label-propagation algorithm used in the disease network is represented by matrix $F_{D}^{\infty }$ (the iteration was stopped when $\left|F_{D}\left(t+1\right)-F_{D}\left(t\right)\right|<10^{-6}$ ).

Finally, the median values of the prediction results $F_{L}^{\infty }$ and $F_{D}^{\infty }$ of the implementation of the label-propagation algorithm in the two networks are used as the estimated scores of the lncRNA–disease association:

$$F^{e}=\frac{F_{L}^{\infty }+{\left(F_{D}^{\infty }\right)}^{T}}{2}$$ (11)

#### Accurate Score of lncRNA–Disease Association

First, the integrated lncRNA similarity matrix $LL^{f}$ is randomly projected in the lncRNA–disease association prediction score matrix $F^{e}$ :

$$LD_{pl}\left(i,j\right)=\frac{LL^{f}\left(i,:\right)\times F^{e}\left(:,j\right)}{\left\|F^{e}\left(:,j\right)\right\|}$$ (12)

In the above formula, $\left\|F^{e}\left(:,j\right)\right\|$ is the 2-norm of $F^{e}\left(:,j\right)$ .

Then, the integrated disease similarity matrix $DD^{f}$ is randomly projected into the lncRNA–disease association estimation-score transposition matrix ${\left(F^{e}\right)}^{T}$ :

$$LD_{pd}\left(i,j\right)=\frac{DD^{f}\left(j,:\right)\times {\left(F^{e}\right)}^{T}\left(:,i\right)}{\left\|{\left(F^{e}\right)}^{T}\left(:,i\right)\right\|}$$ (13)

Finally, $LD_{pl}$ and $LD_{pd}$ are synthesized to obtain the final prediction score.

$$LD^{*}=\frac{LD_{pl}+LD_{pd}^{T}}{2}$$ (14)

## Results

### Parameter Selection Method

**FIGURE 2:**
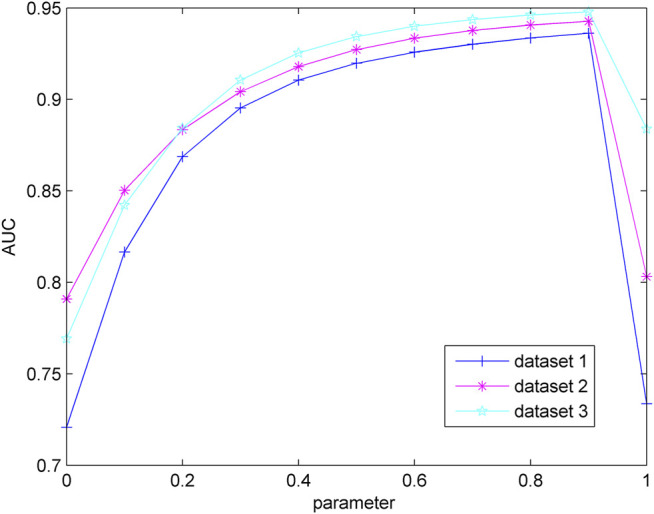
Influence of parameter variation on model prediction accuracy.

In the process of label propagation, each node retains the information of its neighbors and receives its initial label information. In formula 7 (formula 9), the parameter α ∈[0,1] ( β ∈[0,1]) is used to control the rate of retaining information from neighbors, and 1-α(1- β ) means the probability of receiving its initial tag information. For simplicity, we set parameter α and β to the same size. When the parameter value changes from 0 to 1, leave-one-out cross-validation (LOOCV) is implemented on the three data sets to identify the optimum parameters. In LOOCV, we use a known lncRNA–disease association as a test sample and the remaining lncRNA–disease associations as a training sample each time. After the model is trained, the true positive rate (TPR) and false-positive rate (FPR) are calculated to draw the receiver operating characteristic (ROC) curve according to the TPR and FPR under different thresholds. The area under the ROC curve (AUC) is used to evaluate the performance of the model, and a larger AUC value means better prediction performance. The ROC curve and AUC value of each parameter are listed in Figure 2. In the three different data sets, LAPRP has the largest AUC value when the parameter is 0.9. Therefore, we set the parameters to 0.9 on the three different data sets.

### Comparison With Other Methods

**FIGURE 3:**
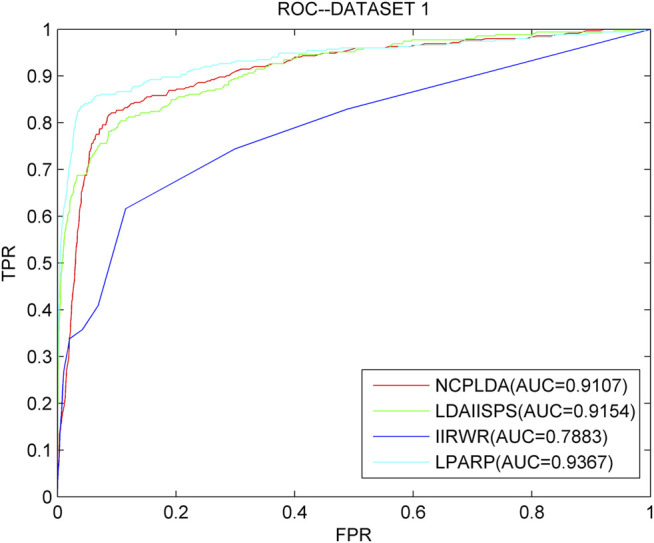
The ROC curves and AUC values of LPARP compared with other methods on the dataset 1.

**FIGURE 4:**
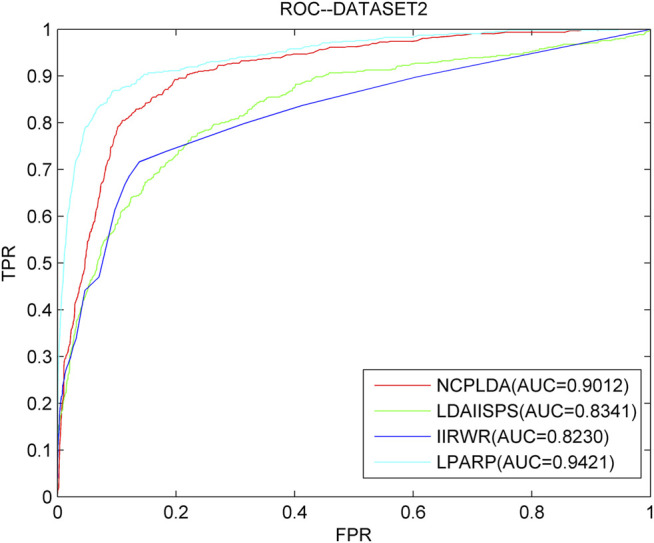
The ROC curves and AUC values of LPARP compared with other methods on the dataset 2.

**FIGURE 5:**
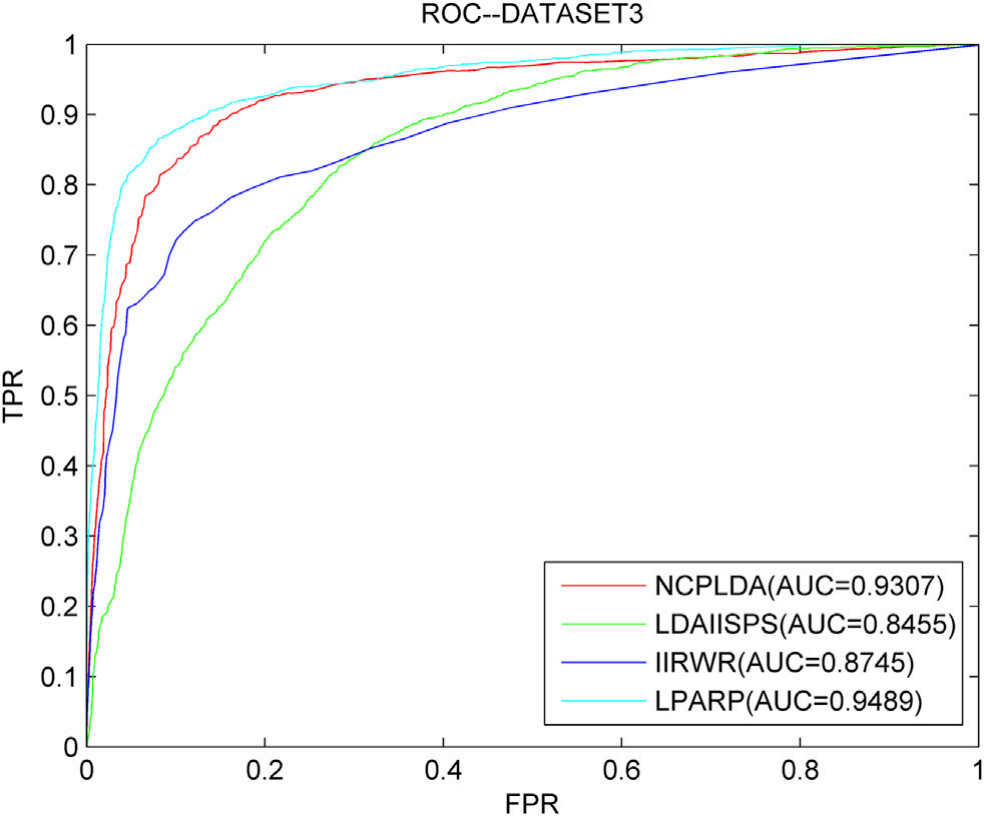
The ROC curves and AUC values of LPARP compared with other methods on the dataset 3.

As we know, NCPLDA (Li G. et al., 2019), IIRWR (Wang L. et al., 2019), and LDAI-ISPS (Zhang et al., 2020) are excellent calculation methods currently used to predict the association of lncRNA diseases. The data used by these three methods is the same as ours. Here, we compare LPARP with them. The comparison results of implementing LOOCV on the three datasets are shown in Figures 3–5.

The AUC values of NCPLDA, IIRWR, LDAI-ISPS, and LPARP on dataset 1 are 0.9107, 0.7883, 0.9154, and 0.9367, respectively, and the AUC values on dataset 2 are 0.9383, 0.9012, 0.8230, 0.8341, and 0.9421, respectively. The AUC values on dataset 3 are 0.9307, 0.8745, 0.8455, and 0.9489, respectively. Obviously, on three different datasets, the prediction performance of LPARP is significantly better than those of NCPLDA, IIRWR, and LDAI-ISPS.

### Prediction for New lncRNAs and Isolated Diseases

With the continuous improvement in lncRNA-recognition technology, more lncRNAs are being unearthed continuously, and most of them have unknown relationships with diseases. We call them new lncRNAs. Isolated diseases refer to diseases without any known relation with lncRNAs. The association prediction of new lncRNAs and isolated diseases helps scientists understand the molecular mechanism of diseases and can help diagnose and treat diseases.

**FIGURE 6:**
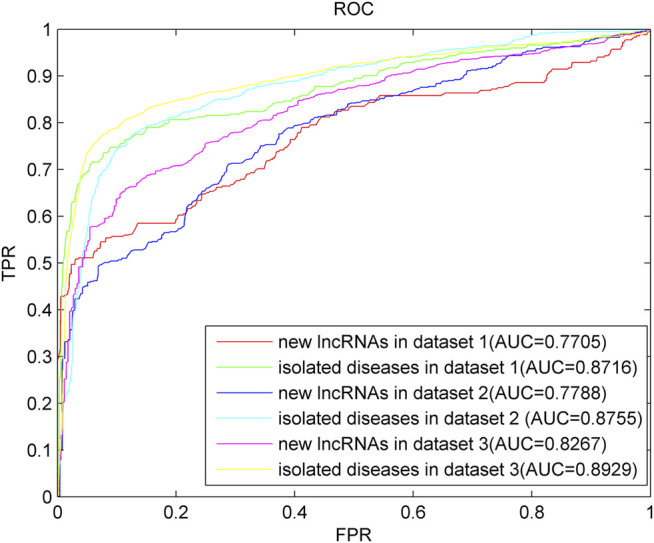
Results of LPARP for new lncRNAs and isolated diseases.

To simulate new lncRNAs, when a certain lncRNA is used as the test sample, all associations between the lncRNA and the diseases are removed. In the experiment, we select each lncRNA as the test sample and all associated information with other lncRNA as the training sample until all lncRNAs are tested as the prediction sample. A similar method is used to verify the prediction effect of LPARP on isolated diseases. For the prediction of new lncRNAs, the AUC on data sets 1, 2, and 3 are 0.7705, 0.7788, and 0.8267, respectively. For the prediction of isolated diseases, the AUC on data sets 1, 2, and 3 are 0.8716, 0.8755, and 0.8929, and the curves are shown in Figure 6. These results indicate that LAPRP has a good predictive effect.

## Case Study

To further evaluate the actual effect of LPARP, the three human diseases including bladder cancer, esophageal squamous-cell carcinoma, and colorectal cancer are selected for the case analysis. The association of dataset 2 is extracted from the lncRNADisease database established in 2015. This database was selected for training, later it was verified in the 2017 lncRNADisease database, which is dataset 3, and the latest related literature.

First, all experimentally verified associations are taken as training samples, and the lncRNA–disease associations that have not been experimentally verified are were taken as candidate associations. For a specific disease, the candidate lncRNAs are sorted according to their prediction scores. For the three diseases bladder cancer, esophageal squamous-cell carcinoma, and colorectal cancer, the top five associations of lncRNA are predicted, as shown in Table 1.

**TABLE 1:** The top 5 lncRNA candidates predicted for bladder cancer, esophageal squamous-cell carcinoma, and colorectal cancer.

disease	lncRNA name	Evidences	Rank
bladder cancer	HOTAIR	LncRNADisease	1
bladder cancer	MALAT1	LncRNADisease	2
bladder cancer	MEG3	Fan et al. (2020)	3
bladder cancer	PVT1	Tian et al. (2019)	4
bladder cancer	GAS5	LncRNADisease	5
Esophageal squamous cell carcinoma	MALAT1	LncRNADisease	1
Esophageal squamous cell carcinoma	MEG3	LncRNADisease	2
Esophageal squamous cell carcinoma	BCYRN1	Unconfirmed	3
Esophageal squamous cell carcinoma	UCA1	Kang et al. (2018)	4
Esophageal squamous cell carcinoma	LSINCT5	Jing et al. (2019)	5
colorectal cancer	MEG3	LncRNADisease	1
colorectal cancer	H19	LncRNADisease	2
colorectal cancer	MINA	Unconfirmed	3
colorectal cancer	UCA1	LncRNADisease	4
colorectal cancer	EPB41L4A-AS1	Bin et al. (2020)	5

Bladder cancer is the ninth most common cancer in the world, and more than 60% of all bladder cancer cases occur in less developed areas of the world (Antoni et al., 2017). Table 1 shows that three of the first five predicted lncRNAs have found supporting evidence in the 2017 version of the lncRNADisease database. MEG3 and PVT1 have not been verified by the lncRNADisease database, but we have manually excavated recent biomedical literature and find them and bladder cancer-related evidence. For example, Fan et al. (2020) found that MEG3 can control the progression of bladder cancer through PI3K/AKT/mTOR pathway regulation. Tian et al. (2019) found that PVT1 can regulate the growth, migration, and invasion of bladder cancer through mir31/CDK1.

Esophageal squamous-cell carcinoma accounts for about 90% of 456,000 cases of esophageal cancer each year (Abnet et al., 2018). The predicted top five lncRNAs are MALAT1, MEG3, BCYRN1, UCA1, and LSINCT5, among which MALAT1 and MEG3 are found to be associated with esophageal squamous-cell carcinoma in lncRNADisease in 2017. Through literature search, UCA1 and LSICT5 are found to be related to esophageal squamous-cell carcinoma. Although we have not manually excavated recent literature to prove that BCYRN1 is related to esophageal squamous-cell carcinoma, we believe that scientists will find the evidence that BCYRN1 is associated with esophageal squamous-cell carcinoma in the future.

Colorectal cancer is the third most common cancer among men and the second most cancer among women (Favoriti et al., 2016). Among the predicted five lncRNAs, three are verified by lncRNADisease database, but MINA and EPB41L4A-AS1 do not show any association with colorectal cancer in the lncRNADisease database. However, Bin et al. (Bin et al., 2020)found in 2020 that EPB41L4a AS1 acts as an oncogene by regulating the Rho/ROCK pathway of colorectal cancer. All of the above literatures were published after the 2017 edition of the lncRNADisease was updated, which confirms the reliability of our method.

To further verify the predictive effect of LPARP on isolated diseases, we select bladder cancer, esophageal squamous-cell carcinoma, and colorectal cancer in dataset2 for case study. The difference between them is that for any kind of disease prediction, to simulate an isolated disease, when training the model, all associations of the disease are removed. The prediction results of the three diseases are shown in Table 2. For Esophageal squamous-cell carcinoma and colorectal cancer, the top five predicted lncRNAs have supporting evidence in the latest lncRNADisease database. For bladder cancer, three lncRNAs have supporting evidence, and MEG3 and PVT1 have not been verified by the lncRNADisease database. When conducting case analysis of common diseases, these two lncRNAs are also considered to be closely related to bladder cancer. Recently, many scientists have proven that they are related to bladder cancer.

**TABLE 2:** The top 5 novel disease-correlated lncRNA candidates predicted for bladder cancer, esophageal squamous-cell carcinoma, and colorectal cancer.

disease	lncRNA name	Evidences	RANK
bladder cancer	HOTAIR	LncRNADisease	1
bladder cancer	MALAT1	LncRNADisease	2
bladder cancer	H19	LncRNADisease	3
bladder cancer	MEG3	Fan et al. (2020)	4
bladder cancer	PVT1	Tian et al. (2019)	5
Esophageal squamous cell carcinoma	HOTAIR	LncRNADisease	1
Esophageal squamous cell carcinoma	MALAT1	LncRNADisease	2
Esophageal squamous cell carcinoma	H19	LncRNADisease	3
Esophageal squamous cell carcinoma	MEG3	LncRNADisease	4
Esophageal squamous cell carcinoma	PVT1	LncRNADisease	5
colorectal cancer	HOTAIR	LncRNADisease	1
colorectal cancer	MALAT1	LncRNADisease	2
colorectal cancer	H19	LncRNADisease	3
colorectal cancer	MEG3	LncRNADisease	4
colorectal cancer	PVT1	LncRNADisease	5

## Discussion

This study shows how to combine lncRNA similarity, disease similarity, and known lncRNA–disease interactions to predict new lncRNA–drug interactions. A new integration method of label-propagation algorithm and random-projection algorithm (LAPRP) is proposed. After evaluating three different datasets, we find that compared with other state-of-the-art methods, LAPRP improves performance effectively and can predict isolated diseases and new lncRNAs. Two types of case studies are carried out on three human diseases: bladder cancer, esophageal squamous-cell carcinoma, and colorectal cancer. The first category is general disease prediction. Among the predicted top five lncRNAs, all five lncRNAs related to bladder cancer, four related to esophageal squamous-cell carcinoma, and four related to colorectal cancer have verified to be the latest confirmation of database or latest literature. The second category is the prediction of isolated diseases. The top five lncRNAs predicted to be related to the three diseases have been confirmed by the latest database or the latest literature. Comparative experiments and case studies show that LAPRP has high prediction accuracy and does not require negative samples. It can be used to predict isolated diseases and new lncRNAs. LAPRP is a useful supplement to experimental methods.

## Data Availability

The original contributions presented in the study are included in the article/Supplementary Material, further inquiries can be directed to the corresponding author.
